# Manipulative therapy and/or NSAIDs for acute low back pain: design of a randomized controlled trial [ACTRN012605000036617]

**DOI:** 10.1186/1471-2474-6-57

**Published:** 2005-11-10

**Authors:** Mark J Hancock, Christopher G Maher, Jane Latimer, Andrew J McLachlan, Chris W Cooper, Richard O Day, Megan F Spindler, James H McAuley

**Affiliations:** 1 Back Pain Research Group, University of Sydney, PO Box 170, Lidcombe, NSW, 1825, Australia; 2 Faculty of Pharmacy, University of Sydney, NSW, 2006, Australia; 3 Discipline of General Practice, Balmain Hospital, 37A Booth St, Balmain, 2041, NSW, Australia; 4 Clinical Pharmacology, UNSW & St Vincent's Hospital, Darlinghurst 2010, NSW, Australia

## Abstract

**Background:**

Acute low back pain is a common condition resulting in pain and disability. Current national and international guidelines advocate general practitioner care including advice and paracetamol (4 g daily in otherwise well adults) as the first line of care for people with acute low back pain. Non-steroidal anti-inflammatory drugs (NSAIDs) and spinal manipulative therapy (SMT) are advocated in many guidelines as second line management options for patients with acute low back pain who are not recovering. No studies have explored the role of NSAIDs and/or SMT in addition to first line management for acute low back pain. The primary aim of this study is to investigate if NSAIDs and/or SMT in addition to general practitioner advice and paracetamol results in shorter recovery times for patients with acute low back pain. The secondary aims of the study are to evaluate whether the addition of SMT and/or NSAIDs influences pain, disability and global perceived effect at 1, 2, 4 and 12 weeks after onset of therapy for patients with significant acute low back pain.

**Methods/design:**

This paper presents the rationale and design of a randomised controlled trial examining the addition of NSAIDs and/or SMT in 240 people who present to their general practitioner with significant acute low back pain.

## Background

Low back pain is a common condition with lifetime prevalence rates reported between 59 and 84% [1]. On any given day 12–33% of people report some back pain [1]. A recent review on the health of Australians placed back problems as one of the three health conditions responsible for the greatest health system expenditure [2]. Not only is back pain common and costly to the health system but an episode of acute low back pain can be seriously disabling and distressing for the patient. In many cases the patient cannot undertake, or is severely restricted in, normal work and home duties. While it is commonly believed that the majority of people with acute low back pain recover spontaneously within 4–6 weeks a recent systematic review of the prognosis of acute low back pain [3] did not find evidence of this. On average, people with acute back pain experience substantial improvements in the first month: pain and disability are reduced by 58% of initial values and 82% of patients have returned to work. Further but smaller improvements occur up to three months, after which pain and disability levels remain nearly constant.

A recent review of international guidelines [4] for the treatment of acute non-specific low back pain showed clear agreement on the appropriate first line of care. Each guideline recommended that the first line of care should be provision of advice and simple analgesic medicines. Advice includes reassuring the patient of a favourable prognosis, encouraging the patient to stay active and discouraging bed rest. It is recommended that medication should be taken in a time contingent manner and paracetamol (4 g daily) is suggested as first line choice due to its low risk of gastrointestinal side effects in otherwise well adults.

Each of the guidelines also recommended that if first line management options provide insufficient pain relief then additional therapies could be considered. However, there is no clear agreement on what the second line management options should include or at what time it/they should begin. All the guidelines endorsed the appropriate use of NSAIDs but only some guidelines (8 of 11) endorsed spinal manipulative therapy (SMT).

Research to date has primarily addressed the issue of whether SMT and/or NSAIDs are more efficacious than placebo, however, this question does not reflect contemporary evidence-based practice in the management of low back pain. For the practitioner following the available guidelines the salient question is whether either of these two treatments are effective when delivered in addition to first line care universally endorsed in the low back pain treatment guidelines.

The recent Australian National Health and Medical Research Council (NHMRC) acute low back pain guidelines 2003 [5] conclude that there is conflicting evidence in this patient group that spinal manipulation provides greater short term pain relief when compared to placebo. Subsequent to the completion of these guidelines, three systematic reviews evaluating the role of SMT in back pain management have been published [6-8]. Each review concludes that spinal manipulation provides a greater reduction in pain and disability than inactive or ineffective therapies, with all trials providing similar estimates for the size and precision of the treatment effect (approximately 18 points of pain relief measured on a 0–100 point scale;95%CI 13–24) [6].

The issue of whether spinal manipulation is more effective than placebo in patients who receive general practitioner (GP) advice and paracetamol as first-line care was not directly investigated in these systematic reviews. However, in the study by Curtis et al [9] all patients received guideline-based care (advice and paracetamol) with half randomised to also receive a course of spinal manipulative therapy. Furthermore, Curtis et al [9] reported that a greater proportion of the manipulative therapy group completely recovered after the first visit compared with the control group: 14% versus 6% (P = 0.01). Patients who received more intense manipulative therapy (four or more treatments) had more rapid return to functional recovery (7.8 days) compared with those who received less treatments (11.1 days; P = 0.02). However this study did not include an inactive manipulation intervention and thus it is possible that the results are influenced by intervention bias as a result of the more frequent patient-practitioner contact in the group who received spinal manipulation.

The Cochrane Review on the role of NSAIDs in the management of low back pain [10] located nine studies comparing NSAIDs to placebo and concluded that NSAIDs were effective for short-term symptomatic relief in patients with acute low back pain. Furthermore, the review concluded that there was strong evidence that various types of NSAIDs are equally effective [10]. Subsequent to this review one study has shown that aceclofenac provides similar results to diclofenac [11] and another study has suggested temporal differences in analgesia produced by diclofenac pain relief when compared to ibuprofen or placebo [12]. However, no studies have examined whether the addition of NSAIDs to standard first-line care produced greater benefit than standard first-line care alone or whether there is an additive effect of manipulative therapy and NSAIDs.

In summary, there is high quality level I evidence that both SMT and NSAIDs are more efficacious than placebo treatments in patients with acute low back pain who receive no additional care. This evidence is of limited use to GPs managing patients with acute low back pain according to the widely accepted first line care. The most important clinical question is whether NSAIDs and/or spinal manipulation are effective when delivered in addition to the first line of care. At present there is no high quality evidence for this. In 2003, the NHMRC clinical practice guidelines for the evidence-based management of acute low back pain identified the need for research into the use of NSAIDs and spinal manipulative therapy and recommended testing these interventions in well-designed randomised controlled trials (RCTs) with 'advice to avoid bed rest and maintain usual activities' as the appropriate comparator [5].

The primary aim of this study is to investigate whether the addition of SMT and/or NSAIDs to GP advice and paracetamol results in shorter recovery times for patients with significant acute low back pain. The secondary aims of this study are to evaluate whether the addition of SMT and/or NSAIDs to GP advice and paracetamol influences pain, disability and global perceived effect at 1, 2, 4 and 12 weeks for patients with significant acute low back pain.

## Methods

This randomised controlled trial will be conducted at approximately 20 general practice clinics and 10 private physiotherapy clinics within Sydney, Australia. Ethics approval has been gained from the University of Sydney Human Research Ethics Committee.

### Study population

Two hundred and forty participants with a new episode of significant acute non specific low back pain who present to GPs will be recruited. GPs will screen potential participants to ensure they satisfy the inclusion and exclusion criteria and will provide participants with an information sheet. The GP will then contact one of the researchers by phone and pass on the patient's contact details. The researchers will then organise an appointment within two days (excluding Sundays) to meet with the participant, formally enrol them in the trial if eligibility is confirmed at which time they will be randomised to a treatment arm of the study.

### Inclusion criteria

To be eligible for the trial participants must meet all of the following criteria as assessed by the GP:

• Primary complaint of pain extending in an area between the 12^th ^rib and buttock crease. This may or may not be accompanied by leg pain.

• New episode of low back pain. This is defined as an episode which was preceded by a period of at least one month without low back pain where the participant was not consulting a health care practitioner or continuing with medication for their low back pain [13].

• Pain of less than six weeks duration.

• Low back pain severe enough to cause moderate pain and moderate interference with normal work including work outside the home and housework (as measured by adaptations of items 7 and 8 of the SF-36).

• No known or suspected serious spinal pathology (metastatic, inflammatory or infective diseases of the spine, cauda equina syndrome, spinal fracture).

• No nerve root compromise evidenced by at least two of the following (i) myotomal weakness, (ii) dermatomal or widespread sensory loss, (iii) hypo or hyper-reflexia of the lower limb reflexes.

• Not currently taking NSAIDs.

• Not currently receiving SMT.

• No spinal surgery within the preceding six months.

• No history of peptic ulcer.

• No allergy to aspirin.

• Not currently receiving anticoagulant therapy.

• No serious co-morbidities preventing prescription of NSAIDs or paracetamol eg: cardiac, liver or renal failure.

• No contraindications to SMT or NSAIDs

### Enrolment and baseline measures

At the first meeting with the researcher, baseline data will be collected from the participant. This will include contact details, personal details, outcome measures, and variables that will be assessed as predictors of response to treatment. Contact details for both the participant and a friend or relative who does not live with them will be collected to optimise follow up rates. Personal details recorded will include age, length of time symptoms have been present and the number of previous episodes of low back pain. The following baseline measures of outcome will be recorded:

• Numerical pain rating scale on a 0–10 scale [14];

• A back specific disability scale (Roland Morris Disability Scale) [15];

• A patient specific measure of disability (Patient Specific Functional Scale)[16];

### Treatment allocation

Immediately after completing baseline measures participants will be allocated into treatment groups. Prior to the start of the study a researcher not involved in data collection or analysis will develop a randomisation schedule and produce consecutively numbered sealed opaque envelopes containing each participant's allocation. Randomisation will be performed using randomly permuted blocks of 4, 8 and 12. The researcher will select the next numerical randomisation envelope and open it. This envelope will contain a plastic bottle with the active or placebo NSAIDs which will be given to the participant along with a consumer medicine information sheet for diclofenac. The participant's name will be fixed to the medication. Placebo tablets with an identical shape and colour (including dose form excipients and coating) are dispensed making it impossible for the researchers or participant to differentiate between the active and placebo NSAIDs. The randomisation envelope will also contain a second smaller envelope containing the participant's allocation into active or placebo SMT. The researcher will give this envelope to the treating physiotherapist to open after the researcher has left. The researcher will therefore remain blinded to allocation for the NSAIDs and SMT arms. Treating physiotherapists can obviously not remain blinded to SMT allocation however they will be trained to respond identically to all patients regardless of treatment group except for the treatment provided.

Patients will be allocated to one of four treatment groups as follows:

• Control group (placebo NSAIDs and placebo SMT)

• NSAIDs group (active NSAIDs and placebo SMT)

• SMT group (placebo NSAIDs and active SMT)

• SMT and NSAIDs group (active NSAIDs and active SMT)

### Treatments

All participants in the study will receive standard care from their GP before baseline and allocation into a treatment group. Standard care in this study will involve advice and paracetamol (1 g four times daily), this being the first line of care advocated in both national and international clinical practice guidelines. Advice will include reassurance of a favourable prognosis and encouragement to avoid bed rest and stay active. Paracetamol will be prescribed at 4 gm per day (Two 500 mg tablets every 6 h). Paracetamol is to be continued at this dosage for a maximum of four weeks. If subjects recover before four weeks (zero or one out of ten pain for seven consecutive days) paracetamol will be stopped. All participants will also receive two follow up visits with their GP, one week and two weeks after their initial visit. At follow up visits GPs will reinforce the initial advice and ensure participants have no adverse reactions to the treatments. GPs will remain blinded to group allocation and instructed not to ask about the physiotherapy management.

Participants allocated to receive active or placebo NSAIDs (diclofenac) will be instructed to take them according to an identical schedule. Dosage will be 50 mg bd taken with food for a maximum of four weeks or until the participant has recovered (zero or one out of ten pain for seven consecutive days).

Participants allocated to receive SMT will receive treatment two or three times per week (at the therapist's discretion) for a maximum of 12 treatments over four weeks. If the subject recovers (zero or one out of ten pain for seven consecutive days) before four weeks the SMT will be stopped. Patients will receive spinal manipulative therapy according to a treatment algorithm developed by the researchers based on the views of expert clinicians and researchers in the field (Additional file 1) [17-20]. The algorithm permits and excludes certain physiotherapy treatments. Consistent with contemporary best clinical practice, the physiotherapist will adjust the treatment to the clinical presentation of the patient rather than apply the same treatment to all patients (as per normal clinical practice). The algorithm however is sufficiently prescriptive to allow replication and accurate description of the trial treatment. All participants will be examined by the physiotherapist who will take a standard history and perform a physical examination that will include assessment of active range of lumbar spinal motion, routine tension tests (straight leg raise, passive neck flexion and prone knee bend), neurological examination (reflexes, muscle strength, sensation) when indicated and the application of manually applied postero-anteriorly directed forces to all levels of the lumbar spine. Based on findings from the examination the physiotherapist will initiate what they consider to be an optimal program of SMT within the guidelines of the study (Additional file 1).

SMT will be delivered by physiotherapists who have postgraduate training in manipulative therapy and who regularly use manipulative therapy in their clinical practice. Participating physiotherapists will be supplied with documentation defining their role in the trial and will undergo one hour of training from a researcher who is a physiotherapist. These physiotherapists will have as minimum training a Graduate Diploma in Manipulative Physiotherapy and two years clinical experience using manipulative therapy techniques.

Therapists will be asked to keep a record of the number of times the participant attended for SMT and details of the treatment including the techniques and dosage. These will be used to ensure compliance with the protocol and to help describe the treatment given in this study.

Placebo SMT will be prescribed using exactly the same schedule as active SMT. The placebo therapy used will be detuned ultrasound (US). The detuned US will be performed in a manner that mimics real US around the area involved for 5–12 minutes. Treatment sessions will last for the same time as for active SMT (30–40 minutes for the initial assessment and treatment and approximately 20 min for follow up sessions). Follow ups will include reassessing the participant's history and physical examination findings however no palpation of the spinal joints will be performed after the initial assessment. A re-evaluation of pain or range of motion will be done after the placebo treatment. In this way we will achieve close matching of the active and inactive interventions in terms of treatment duration and patient/therapist contact.

Patients will be asked not to seek other treatments for their low back pain during the treatment period. In cases where this is unavoidable a record of additional treatments will be kept (and these patients may be excluded from the per protocol but not intention to treat analysis depending on the nature of the treatment). Several mechanisms will be used to ensure that the trial protocol is consistently applied. Protocol manuals will be developed and all involved researchers (general practitioners, manipulative physiotherapists, trial manager and outcome assessors) will be trained to ensure that screening, assessment, random allocation and treatment procedures are conducted according to protocol. An independent researcher will monitor adherence to assessment, randomisation and treatment procedures in a random group of participants.

After four weeks all interventions will cease and participants will be asked not to seek other treatment before the three month follow-up if possible. Participants who do have further treatment will be asked to record the type and amount received.

### Outcome measures

Outcome measures will be recorded by an assessor blinded to group allocation. Outcome measures will be collected using both a daily pain diary completed by the participants and weekly phone follow-ups for the first four weeks and at three months.

The primary outcome is the number of days to recovery, with recovery defined in two ways. Firstly recovery is defined as a pain score of 0 or 1 on a 0–10 pain scale (numerical pain rating scale) that is maintained for seven consecutive days. Secondly recovery is defined as the first day that the patient has a pain score of 0–1 on a 0–10 pain scale. To ensure a precise estimate of the time to recovery, subjects will complete a daily pain diary (completed either first thing in the morning or last thing at night determined by the participant) until recovery. To minimize potential for lost data pain scores from the diaries will be read to the researcher at each of the phone follow ups. The researcher will then transcribe these into a second participant record. Diaries will be kept until the patient has scored 0 or 1 out of 10 for seven consecutive days or for a maximum of three months.

The secondary outcomes are pain (numerical pain rating scale) [21], disability (Patient Specific Functional Scale[16] and Roland Morris Disability Questionnaire [15]), global perceived effect and satisfaction/beliefs about treatment [22]. Secondary outcomes will be recorded at baseline, 1 week, 2 weeks, 4 weeks and 3 months.

Compliance with physiotherapy will be recorded by the treating physiotherapists. Compliance with the medications will be assessed by collecting unused medications at the end of the treatment period (4 weeks).

### Data analysis

Data will be analysed by a statistician who is blinded to group status. The primary analyses will be by intention-to-treat and we will restrict the number of analyses in order to reduce the possibility of Type I errors. For primary outcomes, a p value of <0.05 will be considered statistically significant. For the secondary outcomes a p value of <0.01 will be considered significant.

For the primary outcome of days to reach recovery we will use survival curves with a log-rank statistic to assess differences between groups [23] and, if required, Cox's regression to assess the effects of treatment (group) status on hazard rates for time to recovery. In the primary analysis, number needed to treat (NNT) and the 95% confidence intervals to reach recovery in three months will also be calculated.

For secondary outcomes we will use a mixed model with group as a fixed factor. In these analyses, if there is a significant difference in secondary outcomes between treatment groups, we will conduct post-hoc analyses to inspect differences in secondary outcome variables at 1, 2, and 4 weeks and 3 months. We will also test for any additive or multiplicative effects between treatments on the outcome variables

### Sample size

Sample size was calculated using equations for survival data. Two hundred and forty participants was determined to provide 80% power to detect a 20% difference in recovery rates between the control and intervention groups with an alpha level of 0.05. These calculations were based on a 50% recovery rate in the control group by three months. These numbers are probably conservative and based on results from our recent prognostic study of over 1000 subjects with acute low back pain. Higher rates of recovery will increase the statistical power. We allowed for 10% loss to follow up.

## Conclusion

We have presented the rationale and design for an RCT examining the effects of SMT and/or NSAIDs on patients with significant acute low back pain. The primary outcome will be days to recovery and secondary outcomes include pain, disability and global perceived effect at 1 week, 2 weeks, 4 weeks and 3 months. The results of this trial will be available in 2007.

## Competing interests

The author(s) declare that they have no competing interests.

## Authors' contributions

MH, CM, JL, AM, CC, RD, MS and JM were responsible for the design of the study. All authors read and approved the final manuscript.

## Pre-publication history

The pre-publication history for this paper can be accessed here:



## Supplementary Material

Additional File 1 Description of active SMT. Click here for file
