# Supplementary material for: Manipulative therapy and/or NSAIDs for acute low back pain: design of a randomized controlled trial [ACTRN012605000036617]
Source: BMC Musculoskelet Disord. 2005 Nov 10;6:57. doi: 10.1186/1471-2474-6-57 (PMC1298305; doi:10.1186/1471-2474-6-57)
Supplement: Additional File 1 — Description of active SMT. [file 1471-2474-6-57-S1.doc]

Appendix 1- Description of active SMT

Techniques: Appropriate techniques to be used in this study can broadly be defined as mobilisation or manipulation procedures which aim to produce motion at the joints of the lumbar spine, thoracic spine, sacroiliac joint, pelvis and hip, and involve forces generated by the physiotherapist. The goal of techniques must be to reduce the lumbar spine symptoms within a session and/or between sessions. There will be a trial register of approved techniques which will be provided to all physiotherapists. If a physiotherapist wishes to use any other techniques they must first contact the researchers to get approval. If the technique is deemed appropriate for the trial based on the above definition it will be added to the trial register.

Examples of treatments which are not to be used include neural mobilisation, exercise therapy, McKenzie exercises (mobilisation techniques are okay), muscle energy, massage, therapeutic modalities such as heat or electrical stimulation and treatments of the C/S or skull.

Re-evaluation: Following the application of a technique the patient’s resting symptoms and or symptoms with movement will be re-evaluated. Depending on the patients response to treatment the following guidelines should be used.

- If the patient has improved the technique can continue to be used and dosage may be progressed if there is a rationale that this may give greater benefits.
- If the patient’s condition has not changed the dosage may be increased or a different technique used.
- If the patients initial symptoms are worsened by a technique then the technique should be stopped or at least performed with a lower dosage and careful re-evaluation performed.

Initial dosage: The following guidelines should be used to help set initial dosage. Beyond this, dosage will be modified based on re-evaluation as above. It is expected that patients will receive between 2 and 15 minutes of actual techniques with no single technique at any given spinal level lasting longer than around 3 minutes. Successful techniques may be repeated. Patients whose symptoms are more easily exacerbated will generally be treated for shorter periods.

- Patients judged to have symptoms that are easily exacerbated will initially receive techniques performed with relatively low dosage. In most cases this will involve grade one or two techniques (early to mid range) delivered to the most symptomatic spinal levels in a position of spinal comfort.
- Patients judged to have symptoms that are not easily exacerbated will initially receive techniques performed with relatively high dosage. In most cases this will involve grade three to five techniques delivered to the most symptomatic spinal levels in positions of restricted spinal motion.
